# The NuRD complex component *p66* suppresses photoreceptor neuron regeneration in planarians

**DOI:** 10.1002/reg2.58

**Published:** 2016-08-18

**Authors:** Constanza Vásquez‐Doorman, Christian P. Petersen

**Affiliations:** ^1^ Department of Molecular BiosciencesNorthwestern UniversityEvanstonIllinois60208; ^2^ Robert Lurie Comprehensive Cancer CenterNorthwestern UniversityEvanstonIllinois60208

**Keywords:** Differentiation, eye regeneration, neoblasts, NuRD complex, p66, photoreceptor neurons, planarian, stem cells

## Abstract

Regeneration involves precise control of cell fate to produce an appropriate complement of tissues formed within a blastema. Several chromatin‐modifying complexes have been identified as required for regeneration in planarians, but it is unclear whether this class of molecules uniformly promotes the production of differentiated cells. We identify a function for *p66*, encoding a DNA‐binding protein component of the NuRD (nucleosome remodeling and deacetylase) complex, as well as the chromodomain helicase *chd4*, in suppressing production of photoreceptor neurons (PRNs) in planarians. This suppressive effect appeared restricted to PRNs because *p66* inhibition did not influence numbers of eye pigment cup cells (PCCs) and decreased numbers of brain neurons and epidermal progenitors. PRNs from *p66(RNAi)* animals differentiated with some abnormalities but nonetheless produced arrestin+ projections to the brain. *p66* inhibition produced excess *ovo+otxA*+ PRN progenitors without affecting numbers of *ovo+otxA*− PCC progenitors, and *ovo* and *otxA* were each required for the *p66(RNAi)* excess PRN phenotype. Together these results suggest that *p66* acts through the NuRD complex to suppress PRN production by limiting expression of lineage‐specific transcription factors.

Regeneration requires precise control of cell differentiation in order to build tissues with appropriate composition matched to those regions damaged by injury. Planarians regenerate all adult tissues using pluripotent adult stem cells of the neoblast population and are a model for studying regeneration and adult cell specification (Rink 2012; Elliott & Sánchez Alvarado 2013; Adler & Sánchez Alvarado 2015). Neoblasts are widely distributed throughout the planarian mesenchyme, are the only known dividing cells in planarians, and can be specifically eliminated by gamma irradiation (Newmark & Sánchez Alvarado 2000). However, despite considerable progress in identifying the lineages relating neoblasts to differentiated tissue production and molecules responsible for those transitions (Wang et al. 2010; Wagner et al. 2011; Forsthoefel et al. 2012; Elliott & Sánchez Alvarado 2013; Adler et al. 2014; Scimone et al. 2014; van Wolfswinkel et al. 2014; Adler & Sánchez Alvarado 2015), the mechanisms that ensure appropriate production of missing cell types and in correct numbers are not yet well understood. In principle, phenotypes of differentiated cell overproduction would be useful for dissecting the regulatory logic of cell specification in regeneration, although few have yet been identified in major regeneration models.

The planarian eye is a simple structure with a well‐described cell lineage and has emerged as a model for detailed studies of cell specification and organogenesis in regeneration (Inoue et al. 2004; Lapan & Reddien 2011). This organ assumes a spheroid morphology composed of two principal cell types arranged at opposite mediolateral poles: photoreceptor neurons (PRNs) that send projections to the brain and pigment cup cells (PCCs) that comprise the optic cup presumed to create shadowing important for sensing the direction of received light (Lapan & Reddien 2011). PCCs specifically express *tyrosinase* that is critical for the first step of melanin biosynthesis, while PRNs express *opsin* needed for photoreception. Anterior PRNs express *soxB*, *smad6/7‐2*, and *eye53‐1* and posterior PRNs express *eye53‐2* and *mpl‐2*, among other genes (Collins et al. 2010; González‐Sastre et al. 2012; Lapan & Reddien 2012), although the significance of this division among the PRNs is at present unclear. Functional and expression analysis have identified transcription factors required individually or jointly for PRN and PCC formation (Lapan & Reddien 2011). *otxA* is required for PRN differentiation, *sp6‐9* and *dlx* are required for PCC differentiation, while *ovo*, *six1/2*, and *eya* are required for both PRN and PCC differentiation (Lapan & Reddien 2011, 2012). Newly differentiating PRNs and PCCs are specified in the mesenchyme posterior to each eye, express lineage‐specific transcription factors, and migrate anteriorly prior to terminal differentiation (Lapan & Reddien 2011). Because *ovo* expression is restricted to the eye lineage and marks both PRN and PCC progenitors, these cells have been proposed to arise from common *ovo*+ progenitors (Lapan & Reddien 2012). PRN and PCC progenitors are responsible for homeostatic maintenance of the eye in the absence of injury and also regeneration of new eyes after decapitation or eye resection (Lapan & Reddien 2011, 2012). Thus, a simple cell lineage is responsible for eye regeneration in planarians.

Chromatin‐modifying complexes are critical regulators of cell differentiation and candidates for controlling the production of specific lineages in regeneration. The nucleosome remodeling and deacetylase (NuRD) complex is a conserved multi‐subunit protein assembly that generally functions as a transcriptional repressor by linking detection of methylated DNA by methyl binding proteins to chromatin remodelers (Mi‐2/CHD3/4) and histone deacetylases (HDAC1/2) (Torchy et al. 2015). *p66* is another core component of NuRD identified through biochemical purifications of both *Xenopus* and mammalian extracts (Brackertz et al. 2002; Feng et al. 2002) and contains a GATA‐type zinc finger domain likely involved in sequence‐specific DNA binding to assist targeting NuRD to specific loci (Feng et al. 2002). NuRD additionally has dynamic functions in regulating the balance between pluripotency and differentiation in mammalian embryonic stem cells (Hu & Wade 2012; Reynolds et al. 2012).

We report here a novel function for *p66* in suppressing planarian PRN formation. Inhibition of *p66* by RNA interference caused a reduction of epidermal progenitor cells and ultimately lysis and death, but resulted in regeneration of unpigmented eyes with approximately normal numbers of PCCs and with elevated numbers of PRNs. The majority of *p66(RNAi)* animals had PRNs with expression abnormalities, such as reduced expression of anterior and posterior PRN genes, but could nonetheless send projections toward the brain. Early after head amputation, *p66(RNAi)* animals had excess numbers of *ovo*+ eye progenitor cells and accelerated expression of *otxA*, indicating that *p66* acts early in the eye lineage to suppress formation of photoreceptor progenitors. This suppressive activity on tissue production appeared restricted to PRNs and was not a general suppression of neurogenesis or differentiation in general. We found a similar dysfunction in eye regeneration after attenuated inhibition of *chd4*, the *mi‐2*‐like planarian homolog (Scimone et al. 2010). Together these results suggest that P66 acting through NuRD has a suppressive function in eye differentiation and that chromatin‐remodeling factors can negatively regulate differentiated cell numbers in regeneration.

## Results and discussion

Using sequence homology we identified a planarian homolog of mammalian *p66*, *Smed‐p66*, that contains a GATA zinc finger domain (Pfam score 3.1e‐5). Planarian *p66* is the closest homolog to both human GATAD2A/p66‐alpha (37% identity, e‐value 4e‐16) and GATAD2B/p66‐beta (39% identity, e‐value 1e‐12) identifiable by BLAST searching the *Schmidtea mediterranea* transcriptome. Prior expression profiling of fluorescence‐activated cell sortedneoblasts determined that this gene is expressed maximally in the X2 population of irradiation‐sensitive 2N cells that contain a population of G1 neoblasts and newly differentiating post‐mitotic progenitor cell types (Fig. S1A) (Labbé et al. 2012). Consistent with this observation, *p66* transcript was reduced several days after elimination of neoblasts by inhibition of planarian *h2b*, a neoblast‐specific histone variant (Solana et al. 2012). The low abundance and broad expression of the *p66* transcript in planarians prevented fluorescence in situ hybridizations to be used to determine whether neoblasts versus differentiating progeny preferentially express this gene. However, we used colorimetric in situ hybridizations to confirm its reduction by 7 days after lethal doses of gamma irradiation that depleted both neoblasts and differentiating progeny but not differentiated cells (Fig. S1B, C). These observations together indicate that *p66* is expressed broadly and probably in planarian dividing cells and/or their early post‐mitotic descendants.

**Figure 1:**
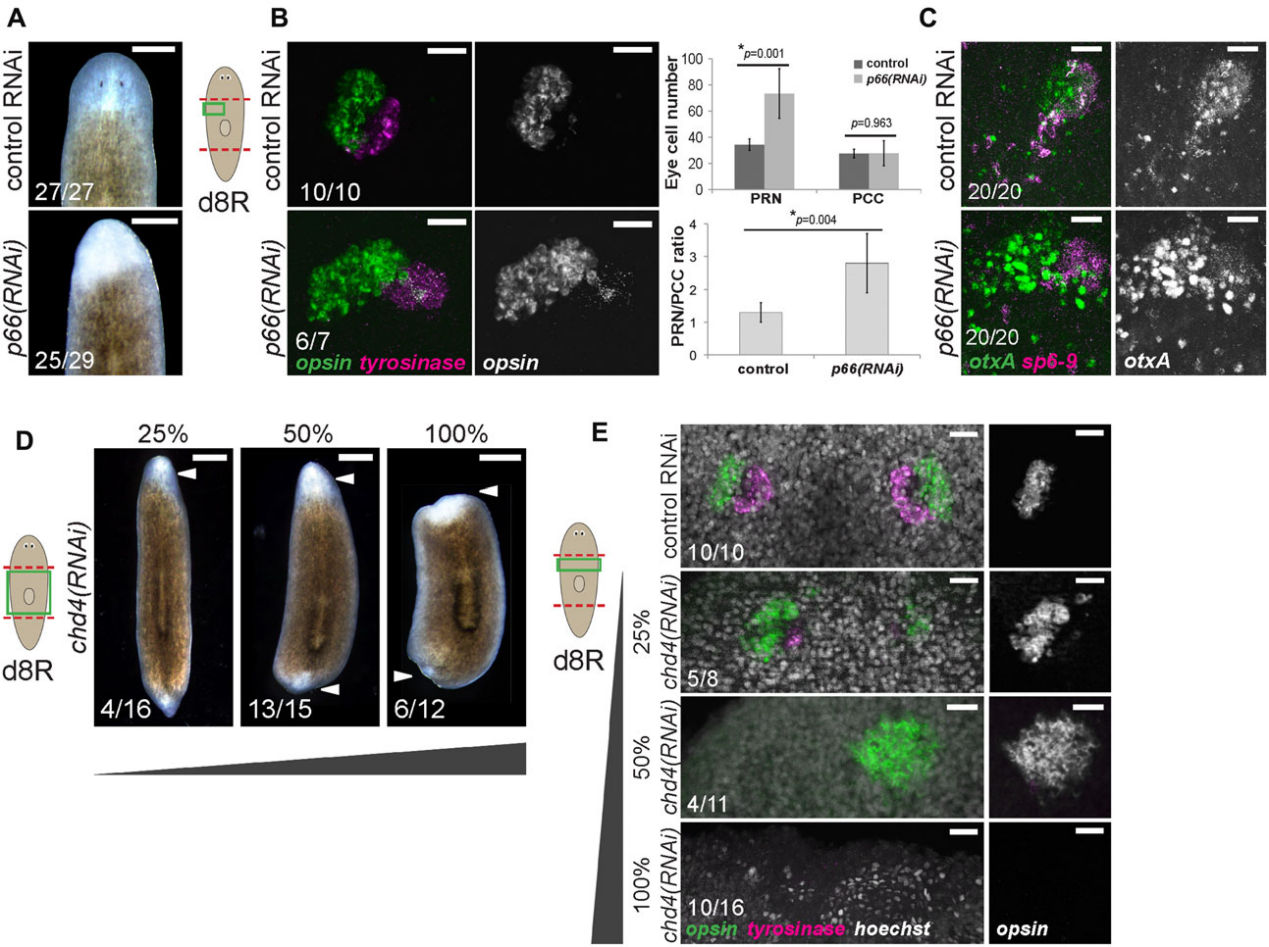
*p66* and the NuRD complex suppress formation of photoreceptor neurons (PRNs) in regeneration. (A)−(C) *p66* RNAi prevented formation of visible eyes but caused production of excess PRNs without affecting numbers of pigment cup cells (PCCs). (A) Images of live animals treated with *p66* RNAi regenerated unpigmented blastemas with apparently no eyes. (B) In situ hybridization to detect PRN marker *opsin* and PCC marker *tyrosinase* after the indicated treatments, with quantification of eye cell numbers and PRN/PCC ratio shown to the right. Error bars show standard deviations from at least 10 eyes for each condition and *P* values were computed from two‐tailed *t* tests. (C) In situ hybridization to detect PRN progenitor marker *otxA* and PCC progenitor marker *sp6‐9* after the indicated treatments. (D)−(E) Attenuated *chd4* RNAi causes production of supernumerary PRNs. *chd4*, encoding a core component of the NuRD complex, is known to be required for blastema formation and survival due to its necessity in producing progenitor cells of the epidermal cell lineage. To circumvent this requirement and investigate possible functions related to eye formation, animals were fed with a mixture of bacteria expressing *chd4* (as indicated) and control dsRNA to produce weakened effects of *chd4* inhibition. (D) Images of live animals treated with *chd4* RNAi failed to regenerate (6/12 animals) or regenerated very small blastemas (6/12 animals). Attenuated *chd4* RNAi dosing formed no eye pigmentation (13/15 50% *chd4* animals, 12/16 25% *chd4* animals), no blastema pigmentation and failed to regenerate tails, reminiscent of effects of *p66* RNAi. (E) Animals treated with 100% *chd4* dsRNA failed to form a blastema or eyes (10/16 animals), but those treated with 50% or 25% dsRNA formed 1–2 eyes with excess PRNs (5/11 did not regenerate, 4/11 one enlarged *opsin*+ cluster, 2/11 two eyes 50% *chd4* animals; and 5/8 two enlarged *opsin*+ clusters and few *tyro*+ cells, 3/8 two eyes 25% *chd4* animals). (A)−(E) Scoring shown in the lower left of the panels indicates the number of animals presenting each phenotype versus total number examined. Cartoons show surgeries (red) and enlarged regions (green). Anterior, top. Bars: 300 μm (A), 25 μm (B, C, E), or 500 μm (D).

In order to investigate *p66* function during regeneration, we performed RNAi and challenged animals to regenerate heads and tails. Inhibition of *p66* caused several defects, including smaller and unpigmented blastemas, lack of visible eyes, tail regeneration failure and ultimately lesions and animal lysis around day 15 (Figs 1A, S2A). In addition, delivery of *p66* double‐stranded RNA (dsRNA) in the absence of injury caused head regression, lesions and lysis, as well as reduction of *p66* transcript abundance (Fig. S2B, C). Prior reports showed that inhibition of other core NuRD components *chd4* (Scimone et al. 2010) or *mbd2/3* (Jaber‐Hijazi et al. 2013) causes regeneration failure and death by interfering with the production of post‐mitotic neoblast descendants fated for epidermal cell formation (van Wolfswinkel et al. 2014). We probed *p66*‐inhibited animals for the presence of neoblasts and epidermal progenitors and found they had reduced expression of a late epidermal progenitor marker (*agat‐1*) and apparently increased expression of a neoblast marker (*smedwi‐1*) but no detectable defect in expression of an early epidermal progenitor marker (*prog‐1*) (Fig. S2D). Similarly, these observations suggest that *p66* positively regulates formation of epidermal progenitors and we did not investigate these effects further.

We were intrigued, however, that eye pigmentation appeared more strongly reduced than blastema formation in *p66(RNAi)* animals (Fig. 1A) and investigated the visual system of these animals in more detail. Surprisingly, despite lacking visible eyes, *p66(RNAi)* animals regenerated twice as many differentiated PRNs expressing *opsin* (Fig. 1B). Thus, planarian *p66* suppresses the production of PRNs formed through regeneration. In addition, although eye cell pigmentation was lost or severely reduced after *p66* RNAi, PCCs expressing *tyrosinase* could be identified in approximately normal location and numbers (Fig. 1B). Likewise, *p66(RNAi)* eye cells contained excess *otxA‐*expressing cells and did not apparently reduce expression of *sp6‐9* (Fig. 1C). Therefore, *p66* does not suppress PRN fates at the expense of PCC fates. Attenuated inhibition of *chd4* also formed excess PRNs and mimicked other aspects of the *p66(RNAi)* phenotype, such as no eye or blastema pigmentation and failure to regenerate tails (Fig. 1D, E), indicating that P66 probably exerts these effects as part of the NuRD complex. We tested the hypothesis that *p66* suppresses formation of neurons in general by examining expression of *gpas*, *cintillo*, *gad*, and *chat*. In all cases, *p66* RNAi reduced the number of brain neurons, as well as brain size (Fig. S3A), indicating that *p66* is not a general inhibitor of neurogenesis. In addition, we used in situ hybridizations to examine the influence of *p66* inhibition on other tissue systems in regenerating animals. The anterior region of *p66(RNAi)* regenerating animals contained *madt‐*expressing intestinal tissue but with some tissue disorganization, and it contained *pou2*/3‐expressing protonephridia‐related tubules though at an apparently reduced density compared to control animals (Fig. S3B). These results together indicate that P66, probably acting with CHD4 as part of the NuRD complex, has a suppressive function in regenerative tissue production that appears limited to PRNs.

**Figure 2:**
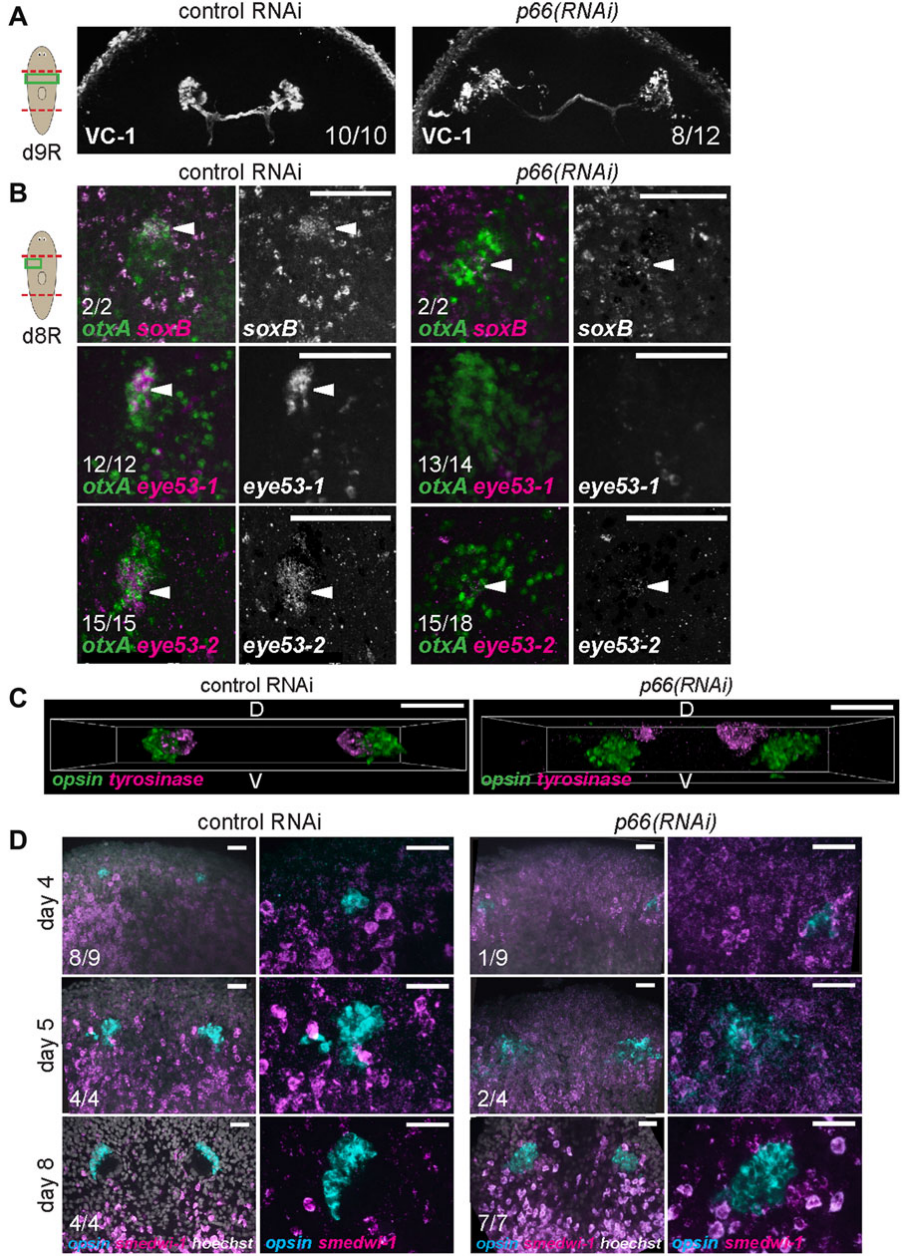
*p66* inhibition causes incomplete terminal differentiation. (A) Photoreceptors from *p66(RNAi)* produce processes that project toward the brain (8/12 animals), as determined by immunostaining with an anti‐arrestin antibody (VC‐1). *p66(RNAi)* animals had projections with normal orientations (8/12 animals), projections disoriented away from the brain (2/12 animals), or no projections (2/12 animals). (B) In situ hybridizations to examine the effect of *p66* RNAi on expression of other genes marking differentiated PRNs. *p66(RNAi)* animals had strongly reduced expression of *soxB, eye53‐1*, and *eye53‐2*. White arrowheads indicate PRN expressing *otxA* and these factors. (C) Transverse views from *z*‐stack confocal projections showing ventral displacement and separation of PRNs with respect to PCCs in *p66(RNAi)* animals. (D) *opsin*+ PRNs from regenerating *p66(RNAi)* animals do not express neoblast marker *smedwi‐1*. *p66(RNAi)* animals showed a delay in PRN (*opsin*+ cells) regeneration; 4 days after head removal, *p66(RNAi)* animals presented no PRN (8/9 animals). Cartoons show surgeries (red) and enlarged regions (green). Anterior, top except in (C) (D, dorsal; V, ventral). Bars: 75 μm (B), 50 μm (C), or 25 μm (D).

We next examined the phenotype of photoreceptor overproduction in more detail to identify the step(s) in differentiation affected by *p66* inhibition. Terminally differentiated PRNs form projections directed toward the brain with projections marked by arrestin protein expression (detected with VC‐1 antibody) (Agata et al. 1998). Furthermore, the anterior PRN domain expresses *soxB* and *eye53‐1*, and the posterior PRN domain expresses *eye53‐2*. Eyes from regenerating *p66(RNAi)* animals had a disorganized appearance but nonetheless the majority of them projected axons labeled by VC‐1 (8/12 animals) (Fig. 2A). However, such eyes had reduced expression of *soxB*, *eye53‐1*, and *eye53‐2* (Fig. 2B). Therefore, although PRNs from *p66(RNAi)* animals show some morphological (VC‐1+) and transcriptional (*opsin*+) signs of terminal differentiation, they lack others. *soxB* RNAi eliminates *eye53‐1* expression without affecting PRN numbers (Lapan & Reddien 2012), making it unlikely that the lack of *soxB* expression in *p66(RNAi)* animals explains their overproduction of PRNs. It is also possible that proper eye morphology or spatial organization is required for expression of *soxB, eye53‐1*, and *eye53‐2*. A transverse view of the eye system showed that PRN and PCC clusters are spatially separated in *p66(RNAi)* animals (Fig. 2C). We additionally tested whether *p66(RNAi)* PRNs retain neoblast character by examining co‐expression of *smedwi‐1* and *opsin*. Like control eyes, *p66(RNAi)* eyes also lacked any *opsin+smedwi‐1*+ cells tested at multiple times in regeneration of a new head (days 4, 5, and 8) (Fig. 2D). Additionally, *p66(RNAi)* animals had decreased body‐wide numbers of mitotic (H3P+) cells, suggesting that PRN overproduction is unlikely due to global neoblast overproliferation (Fig. S3C). Taken together, *p66* probably suppresses PRN production at a step prior to terminal eye differentiation.

**Figure 3:**
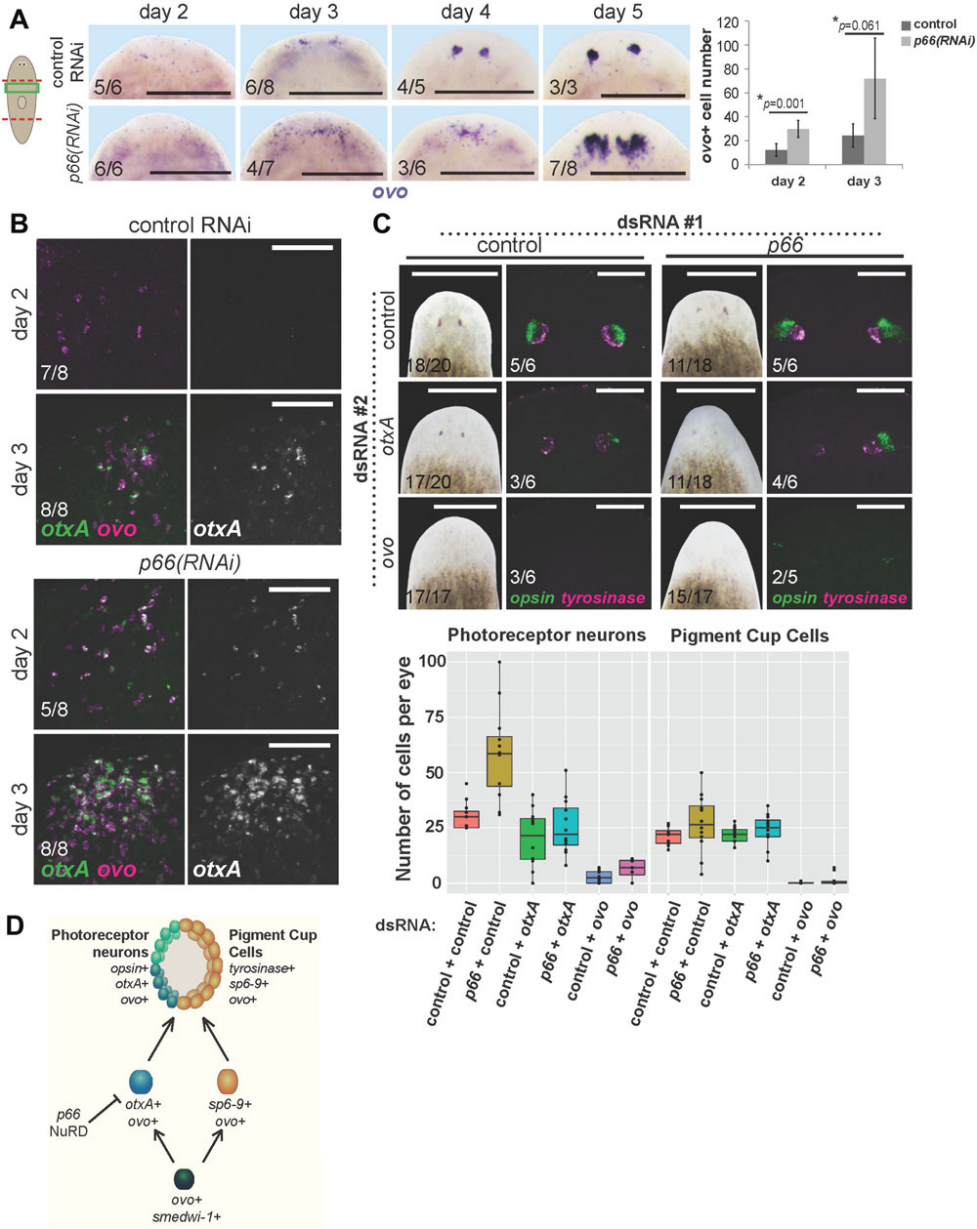
*p66* influences eye differentiation by limiting numbers of *ovo*+ and *otxA*+ eye progenitors. (A) In situ hybridization to detect *ovo* expression during regeneration after *p66* or control RNAi, with quantification of cell numbers at days 2 and 3 shown on the right. Error bars are standard deviations and *P* values are computed from a two‐tailed *t* test. At least three animals were scored for each condition. Numbers of *ovo*+ cells are elevated early in regeneration by day 2–3. (B) Double FISH to detect *ovo* and *otxA* expression early in regeneration. *otxA*+ cells appear earlier in regeneration of *p66(RNAi)* versus control RNAi animals and *otxA* expression is stronger in *p66* RNAi animals. Two days after head removal, control animals presented no or low *ovo* expression (5/8 animals), or two *ovo*+ cell clusters (3/8 animals); none of them expressed *otxA*. In contrast, *p66(RNAi)* animals presented several (3/8 animals) or many (5/8 animals) *ovo*+ cells, and some of these animals weakly expressed *otxA* (4/8 animals). (C) *otxA* or *ovo* RNAi suppresses the *p66(RNAi)* extra PRN phenotype. Animals were injected with pairwise combinations of control, *p66*, *otxA*, and *ovo* dsRNA as indicated for 3 days, then amputated to remove heads or tails, injected with dsRNA again 12 days later, then scored at day 8 for appearance of pigmented eyes, and stained by double FISH for expression of *opsin* and *tyrosinase*. Numbers of PRNs and PCCs were counted in at least 10 eyes and displayed in the boxplots. (D) Model of eye differentiation showing *p66* and the NuRD complex as acting to suppress numbers of *otxA+ ovo*+ photoreceptor neuron progenitors and, consequently, numbers of mature PRNs. Cartoon shows surgeries (red) and enlarged regions (green). Anterior, top. Bars: 400 μm (A), 75 μm (B), 500 μm (C, left), or 100 μm (C, right).

We next examined requirements for *p66* in early specification of eye progenitor cells in decapitated animals that form a new set of eyes through regeneration. *p66(RNAi)* animals had increased *ovo* expression and increased numbers of *ovo*+ cells by days 2–3 of regeneration and a delay in forming *ovo*+ clusters in the eye region (days 3–4), indicating that *p66* affects an early step in eye differentiation (Fig. 3A). Additionally, *otxA* was expressed earlier in regeneration of *p66(RNAi)* versus control animals and accumulated to a higher abundance in regeneration (Fig. 3B). Therefore, *p66* suppresses numbers of *ovo*+ and *otxA*+ cells, suggesting that the expanded production or proliferation of PRN progenitors accounts for the increased formation of PRNs after *p66* inhibition.

Some regulatory genes in planarian regeneration are required specifically for recovery from injury, but others are also required for ongoing tissue replacement in the absence of wounding (Reddien et al. 2005b; Wenemoser et al. 2012). Prolonged inhibition of *p66* in the absence of injury increased numbers of PRN progenitors (*ovo*+*otxA*+ cells) without affecting PCC progenitor numbers (*ovo*+*otxA*− cells) (Fig. S4A). Consistent with these results, eye removal in *p66(RNAi)* animals resulted in increased staining for *ovo+otxA*+ PRN progenitors in the vicinity of both the injured and uninjured eye (Fig. S4B). We conclude that *p66* inhibits formation of PRN progenitors not only during regeneration but also during homeostatic tissue maintenance.

To further examine this model, we used double RNAi experiments to determine the functional relationships between *p66* and two transcription factors required for PRN production, *ovo* and *otxA*. *p66(RNAi);ovo(RNAi)* regenerating animals lacked *opsin*+ and *tyrosinase*+ cells, suggesting that *ovo* is required for the PRN overproduction phenotype when *p66* is inhibited. Similarly, *otxA(RNAi)* suppressed the *p66(RNAi)* extra PRN phenotype (Fig. 3C). These observations suggest that *p66* might either suppress expression of *otxA* and *ovo* in a direct manner or act upon cells expressing these two factors.

These observations eliminate several possible models of action for *p66* in photoreceptor formation. First, *p66* does not suppress PRN fates at the expense of PCC fates, because *p66* RNAi increased PRN numbers without majorly affecting numbers of PCCs. Similarly, *p66* does not suppress formation or proliferation of all *ovo*+ cells, inconsistent with its action on a putative *ovo*+ common progenitor of both PRN and PCC precursors. Finally, *p66* does not simply promote the transition between PRN progenitors and fully differentiated PRN cells because *p66* inhibition elevated the size of both progenitor and differentiated cell populations. Instead, our data are consistent with at least two possible models, that *p66* could either normally suppress proliferation of PRN precursors or promote differentiation of some other cell type at the expense of the PRN lineage. This second model necessitates that alternate cell types diverted in specification due to *p66* inhibition nonetheless require *ovo* and *otxA* function. The NuRD complex is generally a transcriptional repressor with known functions in differentiation; thus we suggest a model in which *p66* and other NuRD factors suppress the differentiation of neoblasts into PRN progenitors, by either directly suppressing expression of *otxA* and *ovo* or acting upon cells expressing these two factors (Fig. 3D). Planarians lack detectable methylated DNA (Jaber‐Hijazi et al. 2013), suggesting that NuRD targeting might be reliant on sequence‐specific DNA binding activity of P66. Future identification of the photoreceptor cell lineages upstream of *otxA* and *ovo* will be important for resolving these models.

Several chromatin‐modifying factors have been found to be necessary for regeneration in general, either for survival or self‐renewal of stem cells or for differentiation into several lineages. In zebrafish, inhibition of the NuRD complex (Pfefferli et al. 2014) or a K27H3 demethylase (Stewart et al. 2009) prevents caudal fin regeneration altogether. In planarians, several major epigenetic regulators have been identified and their functions investigated using RNAi. Planarian viability and neoblast maintenance requires histone methyltransferases *Setd8* (Wagner et al. 2012) and *set1* (Hubert et al. 2013; Duncan et al. 2015), histone methyl binding protein *HP1* (Zeng et al. 2013), and PAF complex component *CTR9* (Onal et al. 2012), suggesting functions for these genes in neoblast self‐renewal, proliferation, or viability. Similarly, PRC2 components *ezh*, *sz12‐1*, and *eed‐1* were required for recovery following sublethal irradiation and expansion of neoblasts in clonal repopulation assays, suggesting a specific role in sustained rounds of self‐renewal (Wagner et al. 2012). By contrast, inhibition of other chromatin modifiers resulted in regeneration failure and animal death without loss of neoblasts, suggesting possible roles in promoting differentiation. BRG1L and SMARCC‐2 (Onal et al. 2012), components of the BAF complex, have this property. Silencing of *chd4/mi‐2* (Scimone et al. 2010), *mbd2/3* (Jaber‐Hijazi et al. 2013), or *RbAp48* (Bonuccelli et al. 2010; Hubert et al. 2015), components of the NuRD complex, caused lethality and regeneration failure without loss of the neoblast population, and *chd4* and *mbd2/3* silencing was shown to prevent production of post‐mitotic progenitors of the epidermal lineage (van Wolfswinkel et al. 2014). However, to date no epigenetic regulators have been implicated in negatively regulating differentiation in regeneration.

*p66* genes are essential for development in both *Drosophila* (Kon et al. 2005) and mouse (Marino & Nusse 2007), consistent with broad organismal functions for the NuRD complex (Basta & Rauchman 2015). However, the NuRD complex can repress the production of particular lineages. For example, *Caenorhabditis elegans mi‐2*/LET‐418 acts with the transcription factor MEP‐1 to suppress germline fate in somatic cells (Unhavaithaya et al. 2002) and mouse Mi‐2beta suppresses production of erythroid progenitors while promoting myeloid and lymphoid progenitors (Yoshida et al. 2008). Our analysis indicates that *p66* and NuRD have repressive functions in eye formation. These observations indicate that, as in embryogenesis, in adult tissue homeostasis and tissue regeneration chromatin‐modifying factors can positively or negatively influence the production of differentiated cells.

## Materials and methods

### Animals and irradiation treatments

The asexual strain of the planarian *Schmidtea mediterranea* was maintained in 18–20ºC planarian water (1× Montjuic salts). Once or twice a week planarians were fed a liver paste and starved for at least a week before experiments. Where indicated, a lethal gamma irradiation dose of 6000 rad was administered using a cesium source; animals were fixed either 3 or 7 days following the irradiation day as indicated.

### Gene cloning, riboprobes and dsRNA synthesis

EST clones of *p66* (EC615093) and *chd4* (EG350668) were cloned into PPR244 vector for dsRNA synthesis and RNAi. Full length *p66* mRNA was identified previously by RNA‐seq transcriptome assembly (dd_Smed_v6_3115_0_1, Planmine [Brandl et al. 2015], also found on SmedGD [Robb et al. 2015]). Riboprobes were synthesized as digoxigenin‐ or fluorescein‐labeled from an antisense polymerase chain reaction (PCR) product using T7 polymerase. The following riboprobes were used in this work: *p66*, *rgs7* (BPKG4258), *smedwi‐1* (Reddien et al. 2005a), *prog‐1* (NB.21.11e) (Eisenhoffer et al. 2008), *agat‐1* (Eisenhoffer et al. 2008), *gpas* (G protein alpha subunit) (Cebrià et al. 2002a, 2002b), *chat* (cholinergic neurons expressing choline acetyltransferase) (Nishimura et al. 2010), *cintillo* (Oviedo et al. 2003), *gad* (glutamine decarboxylase) (Nishimura et al. 2008), *ovo*, *otxA*, *sp6‐9*, *opsin*, *tyrosinase*, *soxB*, *eye53‐1*, *eye53‐2* (Collins et al. 2010; Lapan & Reddien 2011, 2012), *madt* (Petersen & Reddien 2011), *pou2/3* (Scimone et al. 2011).

For RNAi by injection, dsRNA was synthesized in vitro from antisense and sense PCR products with T7 flanking sequences. Silenced genes by injection correspond to *unc‐22* (negative control), *p66*, *chd4*, *ovo*, and *otxA*. For RNAi by feeding, dsRNA‐expressing bacteria were mixed with liver paste and fed to the worms (Reddien et al. 2005a). Silenced genes by feeding were *unc‐22* (negative control), *p66*, and *chd4*.

### RNAi treatments and eye resection

For regeneration RNAi treatment, animals were fed *Escherichia coli* expressing dsRNA mixed with liver paste three times over a week, then cut at least 1 h after the last feeding and analyzed when indicated. For homeostasis RNAi treatment, animals were fed dsRNA bacterial food five times over 3 weeks and fixed 15 days after the first feeding. For double RNAi treatment, animals were injected two to three times with 30 nL of 3 μg/μL dsRNA (final concentration) for three consecutive days. Then, heads and tails were amputated on the third injection day and trunks were allowed to regenerate for 12 days before injecting and cutting again for a second regeneration round. dsRNA concentrations were normalized 1:1 using control dsRNA so that every animal received equivalent doses.

For eye resection, animals were fed dsRNA bacterial food three times over 1 week, and one eye was poked out on day 7 after the first feeding. Then, worms were fed two more times before fixation on day 9 after eye poking. To control for the effect of eye resection, animals from the same batch were not poked. Eye resection was performed on ice‐cooled animals pressing down with an injection needle with a wide opening.

### In situ hybridization and immunofluorescence

Colorimetricwhole‐mount in situ hybridizations (WISH) and fluorescent in situ hybridizations (FISH) were performed using modifications of the Pearson et al. (2009) or King (2014) protocols. In brief, animals were killed in 5% or 7.5% *N*‐acetyl‐cysteine. Animals were fixed in 4% formaldehyde solution, permeabilized and reduced with 0.5% sodium dodecyl sulfate, 1% NP‐40 and 50 mmol/L dithiothreitol at 37ºC, dehydrated in 50% methanol and bleached on a light box overnight in 6% H_2_O_2_ in methanol or for 2 h in 1.2% H_2_O_2_ in 5% formamide. Animals were treated with proteinase K, hybridized overnight at 56°C with digoxigenin‐ or fluorescein‐labeled riboprobe(s), blocked in either 10% horse serum in MABT (maleic acid buffer containing Tween 20) or 5% horse serum, 5% western blot blocking reagent (Roche, Basel, Switzerland) in TNTx, incubated with the corresponding antibody (1:4000 anti‐Dig‐AP, 1:2000 anti‐dig‐POD or 1:2000 anti‐fluorescein‐POD, Roche) in blocking solution and developed with NBT/BCIP (WISH) or tyramide‐fluorophore amplification (FISH). Hoechst 33342 (1:500, Invitrogen) was used as a counterstain for FISH. Animals were mounted in glycerol (WISH) or Vectashield (FISH) prior to visualization.

Antibody staining was performed by fixing animals as above and incubating with VC‐1 monoclonal antibody to detect arrestin protein (a kind gift of R. Zayas) or by fixing animals with HCl and Carnoy's solution (60% ethanol, 30% chloroform, 10% glacial acetic acid) (adapted from Umesono et al. 1997) and incubating with H3P monoclonal antibody to detect phospho‐histone H3. In brief, animals were rehydrated in 50% methanol, rinsed with PBSTB (1× phosphate‐buffered saline, 0.25% bovine serum albumin, 0.3% Triton X‐100), incubated overnight at room temperature with 1:10,000 mouse VC‐1 antibody (Agata et al. 1998) or 1:3000 rabbit H3P antibody (Cell Signaling, Technologies, Danvers, MA, D2C8) solution in PBSTB, rinsed with PBSTB, incubated with horseradish peroxidase conjugated secondary anti‐mouse antibody diluted 1:1000 or anti‐rabbit antibody diluted 1:300 in PBSTB overnight at room temperature, rinsed with PBSTB, and developed for 1 h in 1:150 tyramide working solution (Alexa Fluor 568, T20914, in amplification buffer with 0.0015% H_2_O_2_), rinsed five times for 5 min in PBSTB and then more than six times for 30 min and mounted in Vectashield prior to visualization.

### Imaging and analysis

Imaging of live animals, colorimetric images and H3P‐stained animals was acquired using a Leica M210F dissecting scope with a Leica DFC295 camera. Live animals were placed in cold 1× Montjuic planarian water and imaged on a black background at 20× magnification. For colorimetric images, samples were placed on a white background at 40× or 50× magnification and cropped in Photoshop CS5 9 (Adobe Systems Inc., 345 Park Avenue, San Jose, CA). For fluorescence images, samples were placed on a black background at 50× magnification and cropped in Photoshop CS5 9. Other fluorescence images were taken under either a Leica DM5500B compound microscope with an Optigrid structured illumination system for optical sectioning or a Leica SPE confocal microscope at 10× or 40× magnification. Images shown are maximum projections of *z*‐stacks with adjusted brightness and contrast and switched to CMYK color scheme using Photoshop CS5.

Imaris x64 7.0.0 (Bitplane AG, Badenerstrasse 682, 8048 Zürich, Switzerland) was used for *opsin*+ and *tyrosinase*+ cell counting. Surface module was used to define either *opsin*+ or *tyrosinase*+ volume on each eye and then Spots module was used to count nuclei inside these volumes. *Z*‐stacks analyzed were taken under a confocal microscope at 40× magnification. ImageJ (ImageJ 1.46r, National Institutes of Health, Bethesda, MD) was used for eye progenitor cell (*otxA*+,*ovo*+; *otxA*−,*ovo*+) and brain cell (*cintillo*+, *gad*+) manual counting from *z*‐stacks acquired at 20× in a compound microscope. ImageJ was also used for manual counting of *ovo*+ cells in single‐plane images of colorimetric in situ hybridizations or in a *z*‐series of images of FISH using the “cell counter” plugin to mark and enumerate each cell as it was scored. ImageJ was also used for automated counting of H3P+ cells from images acquired at 50× on a dissecting microscope using the ITCN plugin to detect and count dark peaks, then normalized to area in square millimeters determined using the “analyze particles” function in threshold‐adjusted images. For all cell counting data, averages of at least four animals and standard deviations are reported.

### qPCR analysis

Tail fragments (five pieces, four replicates) were homogenized at day 8 of regeneration in Trizol reagent (Ambion) with a tissue homogenizer (IKA T18 basic ultra‐turrax). Total RNA was extracted using RNeasy Mini Kit (Qiagen) and DNase‐treated (TURBO DNAse, Thermo Fisher Scientific). cDNA was synthesized using reverse transcriptase (MultiScribe, Applied Biosystems) and oligo dT primers (Qiagen). Detection of *p66* mRNA levels was performed by qPCR (Agilent Technologies Stratagene Mx3005P) using EvaGreen 2× qPCR MasterMix (Bullseye, BEQPCR‐S) and the following primers: 5′‐CAGGGCATCATCATCAAACA‐3′ and 5′‐TATTTGATGGCCGATGTGAA‐3′ (*Smed‐p66*), 5′‐GACTGCGGGCTTCTATTGAG‐3′ and 5′‐GCGGCAATTCTTCTGAACTC‐3′ (*Smed‐clathrin*, normalizing gene).

## Supporting information

Additional Supporting Information may be found in the online version of this article at the publisher's website:

Additional Supporting Information may be found in the online version of this article at the publisher's website:
**Figure S1**. *p66* expression is irradiation sensitive.
**Figure S2**. *p66* inhibition causes regeneration dysfunction and death by interfering with production of epidermal progenitors.
**Figure S3**. *p66* does not suppress formation of all neurons or other tissues.
**Figure S4**. Effect of *p66* inhibition on photoreceptor neurons is independent of large tissue removal.
