# Supplementary material for: The NuRD complex component p66 suppresses photoreceptor neuron regeneration in planarians
Source: Regeneration (Oxf). 2016 Aug 18;3(3):168–78. doi: 10.1002/reg2.58 (PMC5011480; doi:10.1002/reg2.58)

Supplemental Figure Legends

Figure S1. *p66* expression is irradiation sensitive

(A) *p66* expression (in FPKM) as measured by a prior RNA-seq study in G2/S/M cells ("X1") enriched for neoblasts, irradiation-sensitive G1/G0 cells ("X2") that contain neoblasts and early postmitotic neoblast progeny, and irradiation-insensitive differentiated cells ("Xins"). Data were compiled from http://planmine.mpi-cbg.de. (B) In situ hybridizations showing broad expression of *p66* in uninjured planarians and reduced expression 3 and 7 days after 6000 Rads of gamma irradiation. (C) Controls showing depletion of neoblasts (expressing *smedwi-1*) and post-mitotic progenitors of the epidermal lineage (expressing *prog-1* or *agat-1)* and little change to differentiated neurons (expressing *rgs7*) in day 3 and day 7 lethally irradiated animals of the same cohort as those in (B). Scoring information included in each panel as number of animals that appeared as shown versus total examined in each condition. Bars, 400 microns.

Figure S2. *p66* inhibition causes regeneration dysfunction and death by interfering with production of epidermal progenitors

(A) Animals were fed *p66* or control dsRNA three times over a week then amputated to remove heads and tails, as depicted in cartoon, and scored eight days later for regeneration defects. *p66* RNAi caused formation of lesions in regenerating head fragments (5/28 animals, yellow arrowhead), which also failed to regenerate tails (20/28 animals) like trunk fragments (25/29 animals). Anterior blastemas failed to form pigment spots in the eye region (25/29 trunk fragments, 26/29 tail fragments). All *p66(RNAi)* animals presented smaller unpigmented blastemas, and ultimately died by lysis. (B) Homeostatic inhibition of *p66* in the absence of injury ultimately caused lethality. Animals were fed bacteria expressing indicated dsRNAs 5 times over two weeks. *p66(RNAi)* caused head regression by day 15 (20/25 animals) and lesions (9/18 animals), and lysis (9/18 animals) by day 21. (C) In situ hybridizations and qPCR to verify reduction of *p66* transcript levels by dsRNA treatment. Top, *p66* was broadly expressed and transcript levels reduced by day 15 of homeostatic RNAi in animals treated as in (B). Bottom, *p66* mRNA was significantly reduced by day 8 of regeneration. Error bars show standard deviations, with 5 animals probed in 4 replicates for each condition and p-values

computed from 2-tailed t-tests. (D) Homeostatic inhibition of *p66* caused a reduction in late *agat-1*+ neoblast progeny and stronger staining of *smedwi-1*+ neoblasts, with no significant changes to early *prog-1*+ neoblast progeny by day 14 of RNAi. Cartoons show surgeries (red) and generated fragments. Bars, 300 (A-B) or 500 microns (C, D).

Figure S3. *p66* does not suppress formation of all neurons or other tissues

(A) In situ hybridizations to examine regeneration of brain neurons after inhibition of *p66* by RNAi. *gpas* expression marks the brain branches, *chat* expression marks cholinergic neurons, *cintillo* expression marks putative chemosensory neurons and *gad* expression marks GABAnergic neurons. Left, *p66* RNAi did not cause production of ectopic *cintillo*+, *gad*+, *gpas*+, or *chat*+ neurons. Right, quantification of *cintillo*+ and *gad*+ cell numbers verifies that *p66(RNAi)* animals produce reduced numbers of these cell types. Error bars show standard deviations, with at least 4 animals probed in each condition and p-values computed from 2-tailed t-tests. (B) Regenerating animals probed for *madt* to label intestine or *pou2/3* to label tubule cells of the excretory system. (C) Proliferation rate assayed by H3P staining that marks mitotic cells. Quantification of H3P+ cells normalized to animal area (mm^2) showed *p66(RNAi)* animals had reduced numbers of mitotic cells by day 8 of regeneration. Cartoons show surgeries (red) and enlarged regions (green). Bars, 100 microns (A), or 400 microns (B, C).

Figure S4. Effect of *p66* inhibition on photoreceptor neurons is independent of large tissue removal

(A-B) In situ hybridizations to detect expression of *ovo* and *otxA* in uninjured animals (A) or after surgical eye removal (B) in control and *p66(RNAi)* animals. (A, right) Inhibition of *p66* in the absence of injury increased numbers of *ovo+otxA+* PRN progenitor cells found posterior of the eye (yellow arrowheads) but did not change numbers of *ovo+otxA-* PCC progenitor cells. Error bars indicate standard deviations, and p-values were calculated from 2-tailed t-tests. (B) *p66* RNAi caused elevation of *ovo*+ cell numbers in both uninjured and resected eyes. *p66(RNAi)* animals were capable of pigmented eye regeneration in absence of head amputation (10/25 animals). Cartoons show surgeries (red) and enlarged regions (green)

Supplementary 1

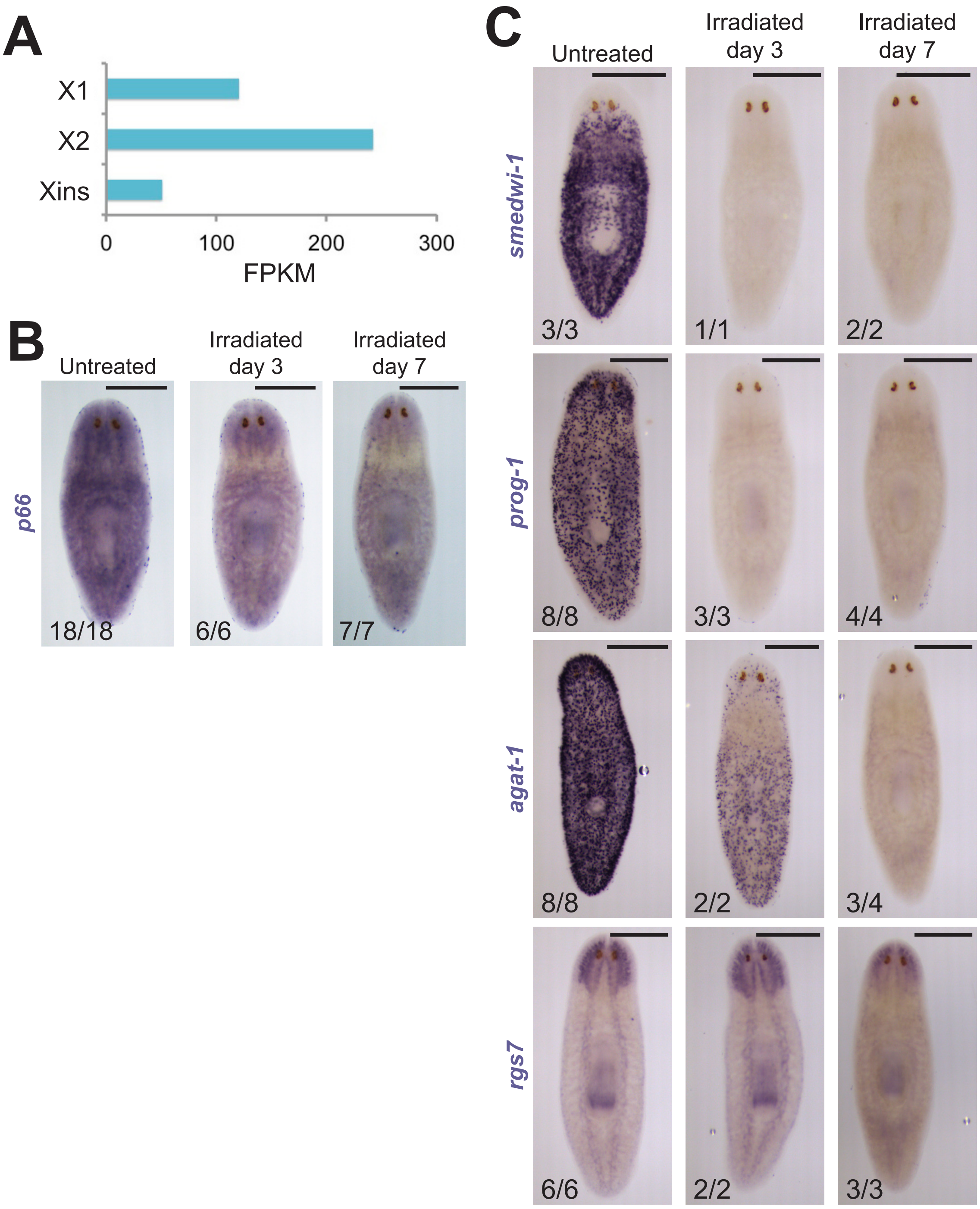

Supplementary 2

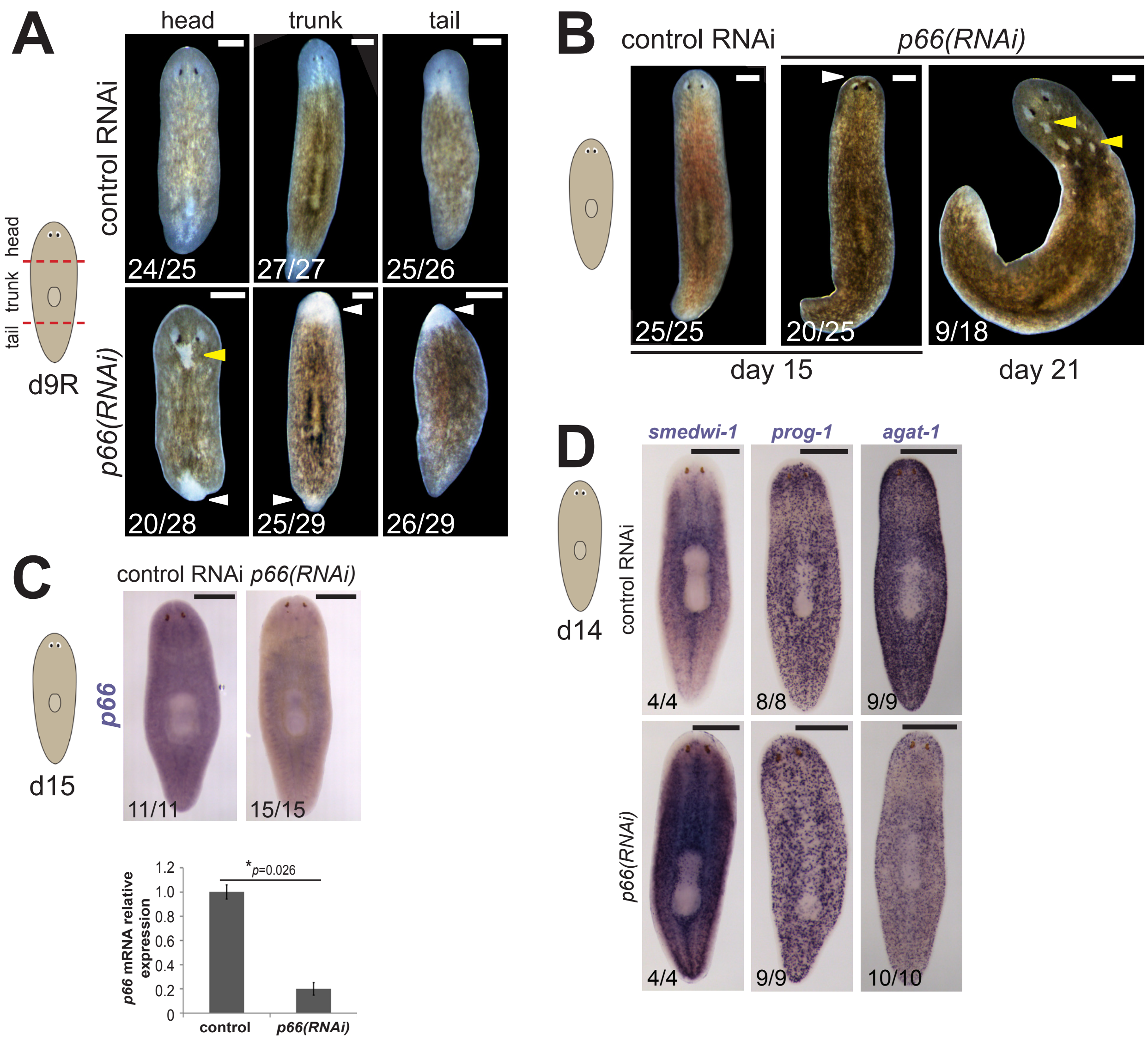

Supplementary 3

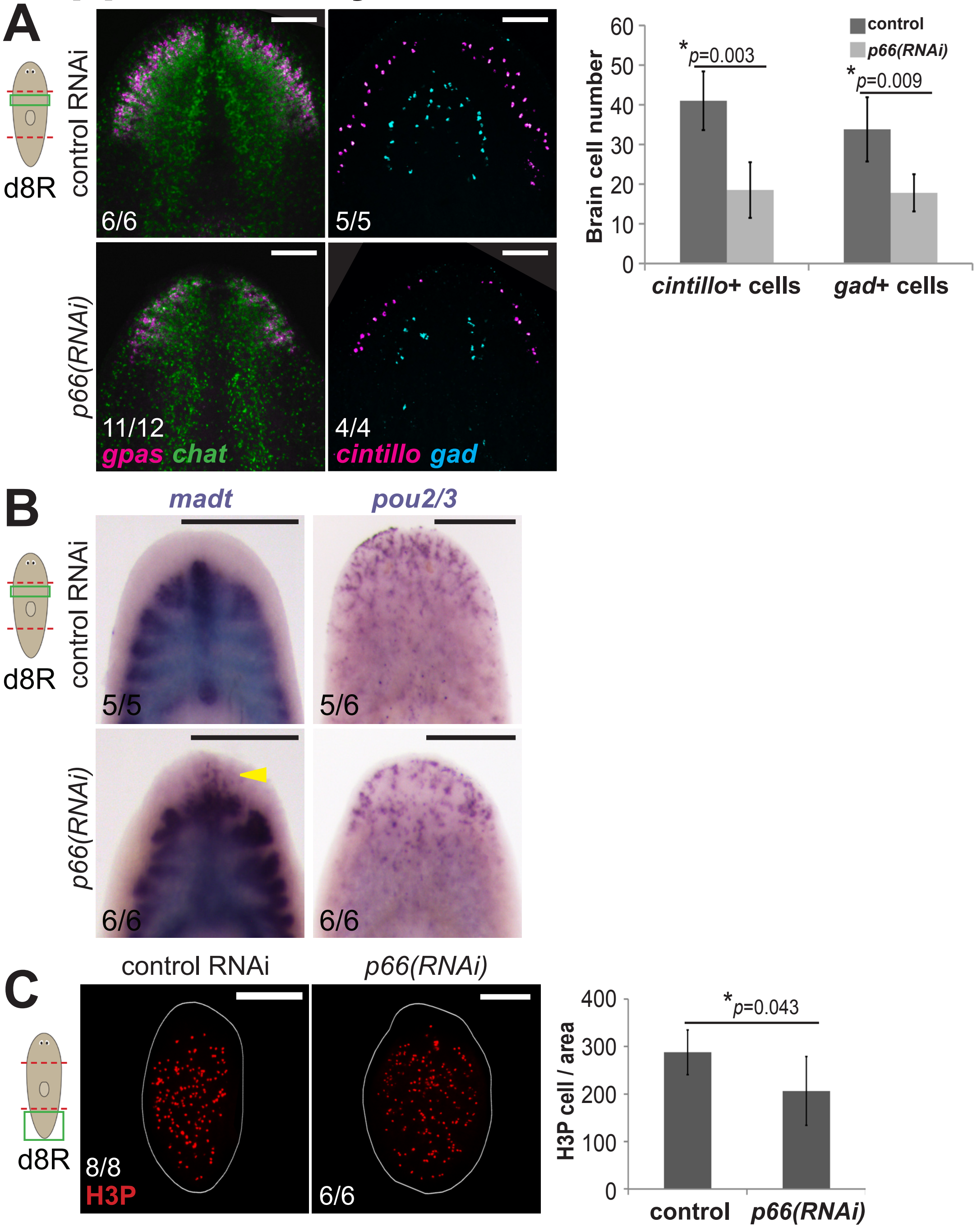

Supplementary 4

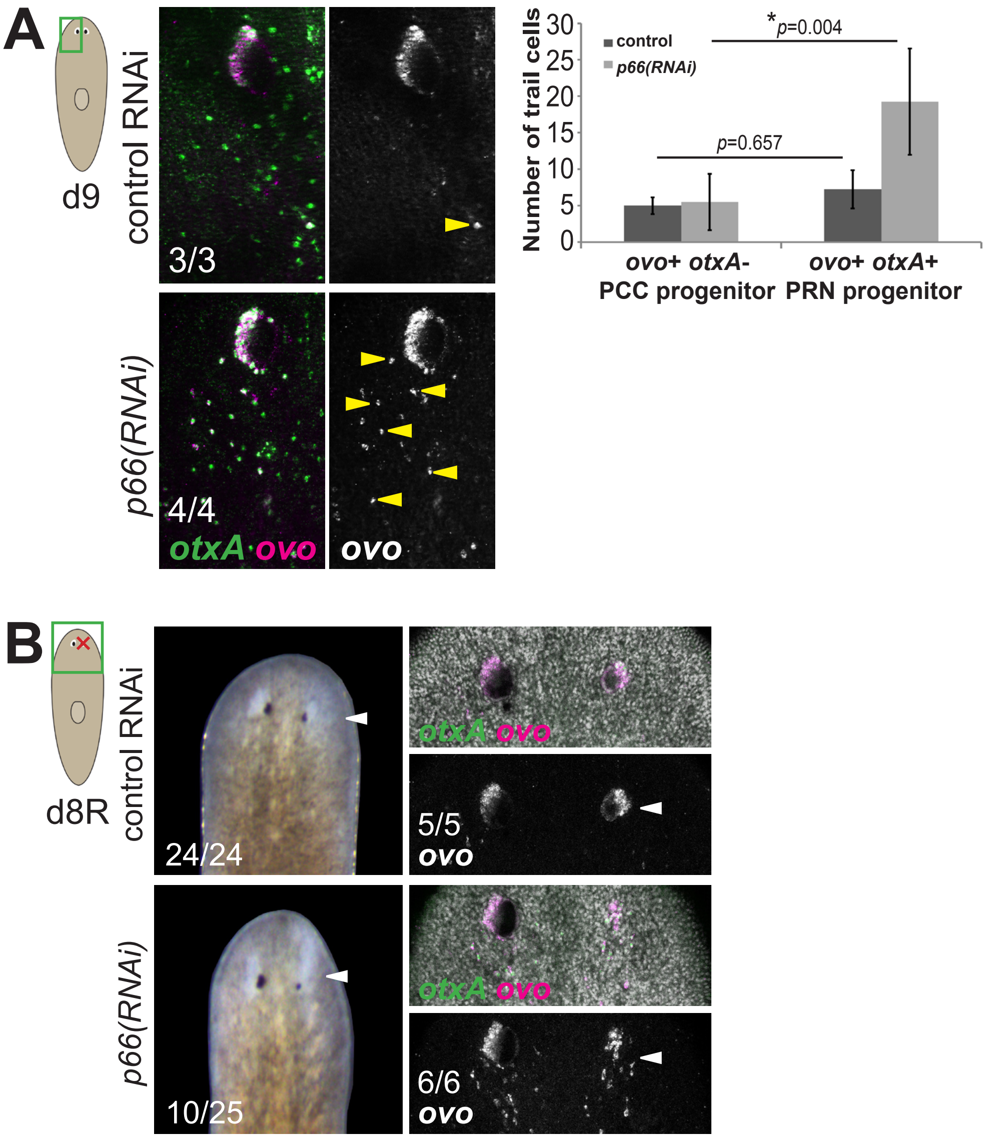

Supplement: Supplementary file 1 — Additional Supporting Information may be found in the online version of this article at the publisher's website: Figure S1. p66 expression is irradiation sensitive. Figure S2. p66 inhibition causes regeneration dysfunction and death by interfering with production of epidermal progenitors. Figure S3. p66 does not suppress formation of all neurons or other tissues. Figure S4. Effect of p66 inhibition on photoreceptor neurons is independent of large tissue removal. [file REG2-3-168-s001.pdf]
